# Exploring the impact of COVID-19 on newborn neurodevelopment: a pilot study

**DOI:** 10.1038/s41598-023-29680-z

**Published:** 2023-02-20

**Authors:** Rosa Ayesa-Arriola, Águeda Castro Quintas, Víctor Ortiz-García de la Foz, Margarita Miguel Corredera, Nerea San Martín González, Nancy Murillo-García, Karl Neergaard, Lourdes Fañanás Saura, Isabel de las Cuevas-Terán

**Affiliations:** 1 University of Cantabria, Santander, Spain; 2 Department of Evolutionary Biology, Ecology and Environmental Sciences (BEECA), Faculty of Biology, University of Barcelona, Institute of Biomedicine of the University of Barcelona (IBUB), Barcelona, Spain; 3 Centro Investigación Biomédica en Red de Salud Mental (CIBERSAM), Madrid, Spain; 4 Mental Illnesses Research Unit, Marqués de Valdecilla Research Institute, IDIVAL, Santander, Spain; 5 Neonatal Unit, Pediatric Service, University Hospital Marqués de Valdecilla, Santander, Spain

**Keywords:** Risk factors, Neonatology, Human behaviour

## Abstract

The COVID-19 pandemic can seize the opportunity to explore the hypothesis of prenatal exposure to viral infections increases the risk for neurodevelopmental disorders. Advancing our knowledge in this regard would improve primary prevention of mental disorders in children. For this pilot study, six-week-old infants born to mothers exposed (n = 21) or unexposed (n = 21) to severe acute respiratory syndrome coronavirus 2 (SARS-CoV-2) were assessed in Santander-Cantabria (Spain) using the Neonatal Behavioral Assessment Scale (NBAS). Groups comparisons were performed to explore the effects that infection and timing of exposure (in terms of the three trimesters of pregnancy). The infants’ competencies and performances on the NBAS were generally similar in the exposed and unexposed to SARS-CoV-2 groups. The most significant difference found was a less optimally response to cuddliness (item on the state regulation domain) particularly in infants born to mothers exposed in the third trimester of pregnancy, and in pull-to-sit (item on the motor system domain). Although our interpretations must be careful, these preliminary results highlight the possible association between prenatal SARS-CoV-2 exposure and poorer development in motor skills and infant interactive behavior. Further longitudinal studies are needed to explore these relationships and disentangle the biological mechanisms implicated.

## Introduction

Prenatal exposure to viral infection has been related to later development of neuropsychiatric disorders^1^. This has been observed after historical pandemics, such as the 1918–1919 Spanish influenza^2^. However, studies addressing the association between prenatal viral infections and mental disorders, such as the viral hypothesis of schizophrenia^3^, have not been able to confirm a causal relationship. In terms of the pandemic of the 2019 Coronavirus Disease (COVID-19), the mechanisms related to the maternal immune response have been proposed to interfere with fetal brain development^4^. The current conditions of the COVID-19 pandemic, including the psychiatric effects, such as worry and anxiety in expectant mothers^5^, and neurological sequelae, such as headache and difficulties with concentration^6^, provide a unique opportunity to determine the associations between fetal exposure to maternal SARS-CoV-2 and postnatal neurodevelopment^4,7^.

Several studies addressing the implications of COVID-19 disease during pregnancy have focused on the effects of the virus on obstetric and delivery complications^8,9^. A recent review indicates that there may be a possible relationship between prenatal SARS-CoV-2 infection and pre-term birth, intrauterine growth restriction and low birth weight^10^. Considering that these neonatal outcomes have been previously associated with a higher vulnerability for later neurodevelopmental disorders^11,12^, children born from SARS-CoV-2 infected mothers could be considered at higher risk along lifetime.

Prenatal infections have been associated with the appearance of unspecific early risk markers, reflected in cognitive, emotional and behavioural deviations in the development of the newborn^13^. Upper respiratory infections during early and middle pregnancy seem to be associated with greater behavioural issues among later offspring^14^. Meanwhile, previous birth cohort studies have identified relationships between maternal immune activation in the third trimester of pregnancy and deviations in the expected behavioural and cognitive scores at 14 months^15^.

The evaluation of neonatal behavior has an enormous value for predicting later developmental trajectories^16^. It has been well established that, in the first weeks of life, newborns’ brains are prepared to actively react to the environment, but also to regulate their inner mental states^17^. Different studies indicate that neonates develop some “rudimentary” abilities for regulating their emotions and their social interactions by integrating sensory, motor and kinetic information^18–20^. The Neonatal Behavioral Assessment Scale (NBAS)^21^ evaluates innate characteristics and behavioral responses in the offspring, with the focus on the interaction with environment and coping strategies.

In this preliminary analysis of a larger longitudinal study conducted in Santander-Cantabria (Spain), we sought to explore the consequences of prenatal exposure to SARS-CoV-2 in early neurodevelopmental outcomes of post-birth offspring. The NBAS scale was used to compare the performance of six-week-old infants that were both exposed (clinical group) and unexposed (control group) prenatally to SARS-CoV-2.

## Materials and methods

### Study design and participants

The present pilot study included infants enrolled in COGESTCOV-19 (Cohort of COVID-19 pregnant women and newborns: study of biological and psychological aspects related to neurodevelopment). All mother-infant dyads were enrolled during pregnancy. For each dyad exposed to SARS-CoV-2 infection during pregnancy, 1 unexposed dyad, defined as the absence of maternal SARS-CoV-2 infection during pregnancy and at delivery, was selected and invited to participate based on infant gestational age at birth and date of birth within a 4-week window. Study procedures approved by the Marqués de Valdecilla University Hospital Review Board (internal code 2020.190). This research was performed in accordance with relevant guidelines/regulations, including the Declaration of Helsinki, and all participants signed an informed consent prior to their inclusion in the study. This pilot study included data from 42 mothers and infants (n = 21 exposed, n = 21 unexposed). Primiparous and multiparous women were included.

### Interviews with pregnant women

Participants in COGESTCOV-19 were reached through midwives and obstetricians who voluntary agreed to inform pregnant women about the research project. In addition, the information was published on social media platforms (Facebook, Instagram and Twitter). In the first visit, prior to both blood test and interview, we obtained written informed consent. Participants were informed that their data and those of their offspring would be kept confidential because all identifiers would be removed from the data to enable anonymity.

For data collection, we used semi-structured interviews to allow for a detailed exploration of the participants’ situations and experiences. During the prenatal interview, mothers were asked for their sociodemographic data and their medical and psychological status. During the postnatal interview, information was obtained regarding their delivery and their infant’s neonatal outcomes. We developed the interview by critically reviewing the literature. We used open ended questions to avoid limiting discussion through prior categorization or by structuring interviews around the researcher’s ideas and assumptions The interview was administrated by expert psychologists.

All of the women in the exposed group showed positive results for SARS-CoV-2 polymerase chain reaction tests. In addition, in terms of the study protocol, blood serological tests were performed in order to confirm the absence of antibodies for SARS-CoV-2 in the control group. Infection severity was categorized as previously described by Gandhi et al.^22^, attending that patients with mild disease typically recover at home, while patients with moderate or severe SARS-CoV-2 infection are usually hospitalized for observation and supportive care.

### Neurodevelopmental assessment in infants

Infants in this pilot study, born between December 2020 and November 2021, were recruited for COGESTCOV-19 as part of a separate protocol to examine the association between prenatal SARS-CoV-2 infection exposure and newborn 6-weeks neurodevelopment.

Infants’ neurobehavioral functioning was evaluated using the Neonatal Behavioral Assessment Scale (NBAS)^21^ at six-weeks old. The NBAS is an scale that assesses the capacity of the newborn to interact and respond to the environment, according to six subdomains: habituation, orientation, motor system, state organization and regulation, autonomic stability and reflexes.

This examination was conducted in a particular sequence according to the authors’ instructions, midway between feedings in a quiet and semi-darkened room with a temperature of 22–27 °C. The NBAS was scored immediately after being performed by a trained, certified and independent reliable examiner. Because the administration of some items required the infant to be in specific states to be administered (e.g., the habituation package should be administered preferably in state 1 (deep sleep) or 2 (light sleep), and the orientation package must be administered while the infant is in state 4 (alert state), we anticipated that some items could not be completed. The test was administrated by a neonatologist and pediatrics expert certified to administrate the NBAS.

### Statistical analysis

Chi-Square and t-tests were used to compare mothers and infant’s information on the prenatally exposed to SARS-CoV-2 (clinical group) and unexposed (control group). Analysis of covariance (ANCOVAs) with mother age, gestational age, age at NBAS assessment and infants sex as covariates were performed to compare clinical and control groups as well as clinical groups attending trimester of pregnancy in the moment of the infection. Post hoc analyses were performed on all main effects (Bonferroni corrected *p* < 0.05). R version 4.1.2 (2021-11-01) was used for statistical analysis. All statistical tests were two-tailed, and significance was set at the 0.05 level.

## Results

This pilot study focused on comparing two groups (prenatally exposed or unexposed to SARS-CoV-2) of 21 infants each, aged 6 weeks, matched 2 by 2 according to mothers age and other controllable sociodemographic information (Table 1). The two groups did not differ with respect to the children’s characteristics, such as gestational age, length and birth weight. Noticeably, mothers did differ on educational level (*p* < 0.001), with lower scores on exposed compared to unexposed.

**Table 1:** Pilot sample characteristics.

	Cases		Controls		Statistic	Value	*p*
	N = 21		N = 21				
	Mean	SD	Mean	SD			
Mother age, y	34.7	3.8	35.1	3.6	t	− 0.378	0.708
Gestational age, w	39.9	1.7	40.0	1.0	W	255.0	0.392
Mother education, y	13.4	4.6	19.6	3.0	t	− 5.136	< 0.001
Weight	3257.9	541.7	3456.9	349.2	t	− 1.415	0.165
Length, cm	49.9	2.1	50.5	2.3	t	− 0.903	0.372
Age at Brazelton assessment, w	6.4	1.5	6.2	0.4	W	208.5	0.771

	N	%	N	%			
Sex (Boy)	12	57.1	10	47.6	X^2^	0.382	0.537
Delivery hospitalization (Scheduled)	5	27.8	4	21.1	Fisher	0.227	0.714
Birth					Fisher	0.734	0.759
Natural	11	55.0	8	42.1			
Induction	6	30.0	8	42.1			
C-section	3	15.0	3	15.8			
Primiparous	14	66.7	14	66.7	X^2^	0.000	1.000
Vaginal delivery	20	95.2	19	90.5	Fisher	0.359	1.000
Full term	19	95.0	19	100.0	Fisher	0.975	1.000
Infection term							
1st	3	14.3					
2nd	8	38.1					
3rd	10	47.6					
Infection severity							
Mild	20	95.2					
Moderate	1	4.8					

The means of both groups were calculated and compared for the 35 items of the NBAS linked to habituation, social interactive, motor system, state organization, state regulation, and autonomic and autonomic nervous system clusters. The infants’ competencies and performances were generally similar in the clinical group and in the control group (Table 2). Statistically significant differences were found for the following items: cuddliness (F = 7.750; *p* = 0.009) (the ability to “let themselves go” in the arms of an adult and relax in a comforting position), which revealed that the unexposed group had significantly higher scores than those in the exposed group; and pull-to-sit (F = 3.678; *p* = 0.063) (hold the head in line with the trunk during the pull-to-sit maneuver) pointing towards higher scores for the unexposed group. Those items respectively belong to state regulation and motor system NBAS domains.

**Table 2:** NBAS items comparisons between cases and controls.

	Cases		Controls		Statistic	Value	p
	N = 21		N = 21				
	Mean	SE	Mean	SE			
Habituation							
1—Response decrement to light	5.8	1.0	6.0	0.9	F	0.042	0.841
2—Response decrement to rattle	3.8	0.9	4.9	0.9	F	0.722	0.407
3—Response decrement to bell	5.2	1.2	5.0	1.0	F	0.023	0.881
4—Response decrement to tactile stimulation of the foot	4.8	1.5	5.7	1.5	F	0.135	0.732
Orientation							
5—Animate visual orientation	4.7	0.6	5.4	0.6	F	0.681	0.415
6—Animate visual and auditory orientation	5.7	0.6	6.6	0.6	F	0.991	0.327
7—Inanimate visual orientation	3.5	0.7	3.5	0.7	F	0.005	0.946
8—Inanimate visual and auditory orientation	5.1	0.6	4.2	0.6	F	1.050	0.314
9—Animate auditory orientation	6.4	0.6	5.7	0.6	F	0.593	0.448
10—Inanimate auditory orientation	6.3	0.5	5.3	0.5	F	2.260	0.143
11—Alertness	5.6	0.6	5.2	0.5	F	0.272	0.606
Motor System							
12—General tone	8.7	0.3	9.2	0.3	F	1.445	0.237
13—Motor maturity	6.2	0.6	6.3	0.6	F	0.038	0.846
14—Pull-to-sit	4.8	0.4	6.1	0.4	F	3.678	0.063
15—Defensive movement	7.1	0.5	6.6	0.5	F	0.515	0.478
16—Activity	7.9	0.6	7.0	0.7	F	0.955	0.335
State Organization							
17—Peak of excitement	5.6	0.6	4.5	0.6	F	1.963	0.170
18—Rapidity of build-up	6.1	0.6	5.9	0.6	F	0.072	0.790
19—Irritability	4.1	0.5	4.8	0.5	F	0.662	0.421
20—Lability of states	5.8	0.6	4.8	0.6	F	1.608	0.213
State Regulation							
21—Cuddliness	4.4	0.3	5.6	0.3	F	7.750	0.009
22—Consolabity	5.1	0.7	4.9	0.6	F	0.025	0.874
23—Self-quieting	5.1	0.6	4.4	0.6	F	0.609	0.441
24—Hand-to-mouth	4.1	0.7	3.8	0.7	F	0.088	0.768
Autonomic Stability							
25—Tremolousness	8.1	0.6	8.3	0.6	F	0.075	0.785
26—Startles	7.0	0.5	6.7	0.5	F	0.255	0.616
27—Lability of skin	6.9	0.6	7.2	0.6	F	0.152	0.698
Smiles							
28—Smiles	2.1	0.4	2.0	0.4	F	0.124	0.727
Supplementary Items							
29—Quality of alertness	5.2	0.5	5.8	0.5	F	0.827	0.369
30—Cost of attention	5.5	0.4	5.0	0.4	F	0.665	0.420
31—Examiner facilitation	5.4	0.4	4.9	0.4	F	0.935	0.340
32—General irritability	5.8	0.5	5.4	0.5	F	0.331	0.569
33—Robustness and endurance	6.6	0.5	5.6	0.5	F	1.581	0.217
34—State regulation	6.9	0.4	7.2	0.4	F	0.553	0.462
35—Examiner's emotional response	7.3	0.5	8.1	0.5	F	1.595	0.215

Using mother age, gestational age, age at NBAS assessment and sex as covariates, and Bonferroni adjusted.

A multiple comparisons analysis was conducted to identify whether the timing of exposure, consisting of the first, second and third trimester of pregnancy, had an effect on neurodevelopment. Results showed that infants exposed to infection on the second trimester of pregnancy presented significantly worse habituation on the response to bell (F = 5.737; *p* = 0.018) (response to a small bell that reflects infants' aptitude for getting used to external stimulation). In addition, those infants exposed to infection on the third trimester of pregnancy presented a poorer response to cuddliness (F = 3.027; *p* = 0.043) (Table 3).

**Table 3:** NBAS items comparisons attending trimester of pregnancy during COVID-19 infection.

	1st Term		2nd Term		3rd Term		Control		Statistic	Value	p	Post-Hoc
	N = 3		N = 8		N = 10		N = 21					
	Mean	SE	Mean	SE	Mean	SE	Mean	SE				
Habituation												
1—Response decrement to light	3.5	2.5	3.6	1.7	8.2	1.4	5.9	0.9	F	1.664	0.210	
2—Response decrement to rattle	0.7	2.5	3.8	1.7	4.9	1.4	4.8	0.9	F	0.892	0.467	
3—Response decrement to bell			2.1	1.3	8.4	1.3	4.9	0.8	F	5.737	0.018	2 < 3 *
4—Response decrement to tactile stimulation of the foot			-4.5	5.6	6.7	1.7	6.0	1.3	F	1.531	0.348	
Orientation												
5—Animate visual orientation	3.4	1.5	4.1	1.0	5.7	0.9	5.4	0.6	F	0.881	0.462	
6—Animate visual and auditory orientation	4.7	1.7	5.0	1.1	6.5	1.0	6.5	0.6	F	0.717	0.550	
7—Inanimate visual orientation	4.3	1.9	2.9	1.2	3.7	1.1	3.5	0.7	F	0.148	0.930	
8—Inanimate visual and auditory orientation	4.7	2.1	5.7	1.1	4.8	1.0	4.3	0.6	F	0.485	0.696	
9—Animate auditory orientation	4.0	1.9	5.5	1.0	7.8	1.0	5.7	0.6	F	1.477	0.244	
10—Inanimate auditory orientation	6.7	1.3	6.5	0.8	6.0	0.8	5.3	0.5	F	0.797	0.506	
11—Alertness	5.8	1.6	5.2	1.1	5.8	0.9	5.2	0.6	F	0.161	0.922	
Motor system												
12—General tone	7.5	0.9	9.1	0.5	8.7	0.5	9.2	0.3	F	1.346	0.276	
13—Motor maturity	5.3	1.8	5.7	1.0	6.9	1.0	6.3	0.6	F	0.283	0.837	
14—Pull-to-sit	5.4	1.3	4.0	0.7	5.4	0.7	6.0	0.4	F	2.036	0.127	
15—Defensive movement	7.4	1.3	6.8	0.8	7.1	0.8	6.6	0.5	F	0.218	0.883	
16—Activity	7.6	1.9	8.1	1.1	7.9	1.0	7.0	0.7	F	0.321	0.810	
State organization												
17—Peak of excitement	7.2	1.6	6.7	0.9	4.2	0.9	4.6	0.6	F	2.215	0.104	
18—Rapidity of build-up	7.6	1.7	6.6	1.0	5.1	1.0	5.9	0.6	F	0.609	0.614	
19—Irritability	3.7	1.6	4.6	0.9	3.9	0.9	4.8	0.6	F	0.364	0.780	
20—Lability of states	7.0	1.6	6.7	0.9	4.6	0.9	4.8	0.6	F	1.633	0.200	
State regulation												
21—Cuddliness	5.0	0.9	4.6	0.5	3.9	0.5	5.7	0.3	F	3.027	0.043	3 < 4 *
22—Consolabity	6.9	2.1	4.8	1.1	4.7	1.0	5.0	0.6	F	0.276	0.842	
23—Self-quieting	5.3	1.7	6.3	0.9	3.7	1.0	4.4	0.6	F	1.422	0.254	
24—Hand-to-mouth	5.2	2.0	4.7	1.1	3.1	1.1	3.8	0.7	F	0.429	0.734	
Autonomic stability												
25—Tremolousness	7.0	1.7	7.6	0.9	8.9	0.9	8.3	0.6	F	0.481	0.697	
26—Startles	8.3	1.4	6.8	0.8	6.7	0.8	6.7	0.5	F	0.432	0.731	
27—Lability of skin	9.1	1.5	7.8	0.9	5.3	0.8	7.3	0.5	F	2.088	0.120	
Smiles												
28—Smiles	3.5	1.1	1.6	0.6	2.1	0.6	2.0	0.4	F	0.860	0.471	
Supplementary items												
29—Quality of alertness	4.9	1.4	4.9	0.8	5.5	0.8	5.8	0.5	F	0.385	0.764	
30—Cost of attention	6.4	1.1	5.1	0.6	5.5	0.6	5.0	0.4	F	0.576	0.635	
31—Examiner facilitation	5.9	1.1	5.8	0.6	4.9	0.6	4.9	0.4	F	0.678	0.572	
32—General irritability	7.1	1.4	6.3	0.8	4.8	0.8	5.4	0.5	F	0.995	0.407	
33—Robustness and endurance	5.9	1.6	6.7	0.9	6.7	0.9	5.6	0.6	F	0.559	0.645	
34—State regulation	5.9	1.1	7.0	0.6	7.1	0.6	7.2	0.4	F	0.466	0.708	
35—Examiner's emotional response	7.3	1.3	8.0	0.7	6.6	0.7	8.1	0.5	F	1.092	0.366	

Using mother age, gestational age, age at NBAS assessment and sex as covariates, and Bonferroni adjusted.

## Discussion

Although the results of this pilot study are preliminary and should be interpreted with caution, they are very interesting and deserve attention. The observation that the performance in the item cuddliness has a lower score in infants whose mothers were exposed to SARS-CoV-2, and more specifically in those exposed during the third trimester of pregnancy, is particularly interesting. From birth, newborns recognize and create structures of social interaction with others that are critical for optimal brain development^23,24^. Social interactions, especially with their caregivers, are based on face-to-face exchanges and close physical contact that lead to attachment^25^. Previous studies have related a less optimal response to cuddliness in infants of depressed mothers, suggesting that those infants were more aroused^26^. It is interesting to note that none of the infants whose mothers were exposed to SARS-CoV-2 in the first trimester of pregnancy completed the item response to bell. This item requires that the children be in state 1 (deep sleep) in order to initiate the administration. This could be due to an excess of excitation that is not triggered by the environment but is residual. One possible interpretation of this detail is that the level of arousal of the cases group exposed during the first and second trimester of pregnancy was higher than in those exposed in the third trimester that were similar to controls, impairing a proper state regulation. In addition, it would be possible that postnatal influences, such as maternal behavior linked to a variety of changes related to SARS-CoV-2 exposure and pandemic situation in general, could also contribute and be particularly relevant for this finding.

Those findings points towards that those infants prenatally exposed to SARS-CoV-2 could present difficulties on state regulation, that in accordance with Belot et al.^27^ could be partially explained by physiological alterations in the nervous system. This higher level of arousal could be associated with greater activation of infants’ hypothalamic–pituitary–adrenal (HPA) axis, and relates to stress exposure during critical periods, which in turn increases the risk on psychopathology^28^. Noticeably, Provenzi et al.^29^ observed alterations in the regulatory capacity of 3-month-old infants whose mothers were pregnant during the COVID-19 pandemic compared to infants whose mother were pregnant previous to this period. This finding highlights the importance of psychosocial stress for regulating children’s HPA axis functioning, especially in the third trimester of pregnancy when this system has already been formed. These results are in line with findings from Shuffrey et al.^30^, who observed social and motor developmental alterations in children born from mothers pregnant during COVID-19 pandemic period, but not in those exposed prenatally to SARS-CoV-2. Biological data, such as cortisol levels obtained from saliva samples in those infants, will be very useful to explore the functioning of their HPA axis.

Another result that worth considering is that of the poorer performance on pull-to-sit item, which is related to head control, in infants whose mothers were exposed to SARS-CoV-2. Vulnerabilities in infants’ fine and gross motor skills may have significant consequences for later motor development^31^. Specifically, poor postural control during pull-to-sit has been suggested as a predictor of developmental disruption^32^. Previous studies have linked childhood motor deficits with the risk of developing autism-like disorders^33^ and adult schizophrenia spectrum disorders^31^. Lower scores in our cases could be indicating a certain motor immaturity or what have been termed neurological soft signs (NSS). NSS entail minor motor and sensory differences, a common feature in children with neuropsychiatric disorders^17,34^, that could be considered a proxy marker for underlying neuropsychological deficits.

This study has several limitations. First, since it is a pilot study, the sample is relatively small (only 21 exposed and 21 unexposed). However, as it is an ongoing project, more participants are being recruited, adding to the current sample in later analyses. It should be noted, however, that sample sizes found in the literature related to the NBAS scale are not much larger than our current sample. This is possibly due to the extensive training required to administer the NBAS scale. Second, none of the cases in the study presented with severe COVID-19 symptoms. This circumstance may either be due to a lack of serious cases in our community at the time, or that the few severe cases that might have occurred suffered too much to be enrolled in a research study of this kind. Third, attending that COVID-19 vaccination safety for pregnant people was not confirmed until august 2021, when most of the women in this pilot study have been already evaluated, information about vaccine status and vaccine attitude will be the considered in coming studies^35^. Finally, pairing was not optimal for years of education since, in line with results reported by Shuffrey et al.^30^, significantly differed between exposed and unexposed women. A possible interpretation is that voluntary participation was related to higher education in the unexposed group while negatively related to enroll in the exposed group. In addition, to the already known unequal distribution of the disease, which in already marginalised populations present higher rates and risks^36^.

## Conclusions

Our findings suggest that prenatal exposure to SARS-CoV-2 infection could be related to alterations in different neurodevelopmental domains, particularly those related to interactive behavior and motor development. The timing of exposure to the infection could play an important role on the severity and type of these deficits. It is essential to continue studying these cohorts of children over time with a particular focus on primary prevention and early intervention.

## Data Availability

The datasets generated during and/or analysed during the current study are available from the corresponding author on reasonable request.
