# Author Correction: Exploring the impact of COVID-19 on newborn neurodevelopment: a pilot study

**DOI:** 10.1038/s41598-024-73343-6

**Published:** 2024-09-20

**Authors:** Rosa Ayesa-Arriola, Águeda Castro Quintas, Víctor Ortiz-García de la Foz, Margarita Miguel Corredera, Nerea San Martín González, Nancy Murillo-García, Karl Neergaard, Lourdes Fañanás Saura, Isabel de las Cuevas-Terán

**Affiliations:** 1 University of Cantabria, Santander, Spain; 2 Department of Evolutionary Biology, Ecology and Environmental Sciences (BEECA), Faculty of Biology, University of Barcelona, Institute of Biomedicine of the University of Barcelona (IBUB), Barcelona, Spain; 3 Centro Investigación Biomédica en Red de Salud Mentall (CIBERSAM), Madrid, Spain; 4 Mental Illnesses Research Unit, Marqués de Valdecilla Research Institute, IDIVAL, Santander, Spain; 5 Neonatal Unit, Pediatric Service, University Hospital Marqués de Valdecilla, Santander, Spain

Correction to: *Scientific Reports*10.1038/s41598-023-29680-z, published 20 February 2023

In the original version of this Article, the Funding section was incomplete.

“This study has been funded by Government of Cantabria (INNVAL20/02). Dr. Ayesa-Arriola is funded by a Miguel Servet contract from the Carlos III Health Institute (CP18/00003), carried out on Fundación Instituto de Investigación Marqués de Valdecilla. Two authors are funded by pre-doctoral research grants, one from the Catalonian authorities to Águeda Castro Quintas (AGAUR-FI_B 00233_2020) and one from the Valdecilla Biomedical Research Institute and the University of Cantabria to Nancy Murillo (BOC49, REF. IDI13). Nerea San Martin González is supported by a research initiation grant from the Institute of Biomedicine of the University of Barcelona. No pharmaceutical company has financially supported the study. This study was supported by the Spanish Ministry of Economy and Competitiveness, Instituto de Salud Carlos III (PI22/01245).”

now reads:

“This study has been funded by the Government of Cantabria (INNVAL20/02). Dr. Ayesa-Arriola is funded by a Miguel Servet contract from the Carlos III Health Institute (CP18/00003), carried out by Fundación Instituto de Investigación Marqués de Valdecilla. Two authors are funded by pre-doctoral research grants, one from the Catalonian authorities to Águeda Castro Quintas (AGAUR-FI_B 00233_2020) and one from the Valdecilla Biomedical Research Institute and the University of Cantabria to Nancy Murillo (BOC49, REF. IDI13). Nerea San Martin González is supported by a research initiation grant from the Institute of Biomedicine of the University of Barcelona. No pharmaceutical company has financially supported the study. This study was supported by the Spanish Ministry of Economy and Competitiveness, Instituto de Salud Carlos III (PI22/01245). Ayuda cofinanciada por el Fondo Europeo de Desarrollo Regional (FEDER) “Una manera de hacer Europa”.

The original Article has been corrected.

